# Multi-objective optimization of MOSFETs channel widths and supply voltage in the proposed dual edge-triggered static D flip-flop with minimum average power and delay by using fuzzy non-dominated sorting genetic algorithm-II

**DOI:** 10.1186/s40064-016-2987-6

**Published:** 2016-08-22

**Authors:** Farshid Keivanian, Nasser Mehrshad, Abolfazl Bijari

**Affiliations:** Department of Electrical and Computer Engineering, University of Birjand, Birjand, Iran

**Keywords:** Optimum MOSFETs channel widths and power supply, Proposed Dual Edge-Triggered Static D Flip-Flop, Minimization of average power and delay, Power delay product, Fuzzy NSGA-II

## Abstract

**Background:**

D Flip-Flop as a digital circuit can be used as a timing element in many sophisticated circuits. Therefore the optimum performance with the lowest power consumption and acceptable delay time will be critical issue in electronics circuits.

**Findings:**

The newly proposed Dual-Edge Triggered Static D Flip-Flop circuit layout is defined as a multi-objective optimization problem. For this, an optimum fuzzy inference system with fuzzy rules is proposed to enhance the performance and convergence of non-dominated sorting Genetic Algorithm-II by adaptive control of the exploration and exploitation parameters. By using proposed Fuzzy NSGA-II algorithm, the more optimum values for MOSFET channel widths and power supply are discovered in search space than ordinary NSGA types. What is more, the design parameters involving NMOS and PMOS channel widths and power supply voltage and the performance parameters including average power consumption and propagation delay time are linked. To do this, the required mathematical backgrounds are presented in this study.

**Conclusion:**

The optimum values for the design parameters of MOSFETs channel widths and power supply are discovered. Based on them the power delay product quantity (PDP) is 6.32 PJ at 125 MHz Clock Frequency, L = 0.18 µm, and T = 27 °C.

## Background

The layout of an electronics circuit plays an important role in the design and usability of many products (Mihajlovic et al. 2007). In computers, communications, and many other systems, the flip-flops are fundamental building blocks. They are the important timing elements in digital circuits which have great impacts over power consumption and speed. The performance of Flip-Flop influence the performance of whole synchronous circuit, particularly in deep pipelined design (Bhargavaram and Pillai 2012). In this study, D Flip-Flop is considered. The optimum layout design of D Flip-Flop can be defined as an optimization problem. That is solved by the Multi-objective Evolutionary Algorithm (MOEA). MOEAs are well-suited for solving several complex multi-objective problems with two or three objectives (Lücken et al. 2014). As the performance of most MOEAs for problems with four or more conflicting objectives is severely deteriorated (Lücken et al. 2014), for this study, we define two conflicting objectives. Here we use a multi-objective evolutionary algorithm based on Genetic Algorithm. The non-dominated sorting genetic algorithm-II, NSGA-II, has questionable exploratory capability (Coello Coello et al. 2007). There are three evolutionary processes such as mutation, crossover, and selection. The mutation operator is used to increase the diversity of off-springs or generated solutions which is inspired by genetic diversity from one generation of population chromosomes to the next. The crossover which is inspired by genetic inheritance in parent children is applied to vary the situation or features of a chromosome or chromosomes from one generation to the next. The selection procedure is done to select the better or more optimum solutions.

In this study, for the proposed problem we will define two objective functions such as average power consumption and propagation delay time. They are minimized by proposed FNSGA-II when its three operators are implemented. For multi-objective optimization we are looking for the series of non-dominated solutions that are placed in the category of Pareto Front. There will not be any other solution better than non-dominated solutions and no solution will dominate them. The solutions of Pareto Front are ranked as the first Front F1 since they are the closest Front to the ideal solution in comparison with the other solutions (Coello Coello et al. 2007).

In sequential circuits there are many Flip-Flops. Since changes in the data inputs of a gated D latch flip-flop have no effect unless the clock is asserted, the propagation delay is not considered when the data inputs are entered (Mohanram 2014). In combinational logic circuits the basic blocks are the gates while in sequential logic circuits the flip flops are principal building blocks. Flip-Flops are clock based devices. Each flip flop can store one bit. D Flip Flop is the best choice in Integrated Circuit design works (Elias 2014). The D flip-flop is also known as a “data” or “delay” flip-flop. It captures the value of the D-input at a definite portion of the clock cycle and then the captured value becomes output Q. The D flip–flip is one of the most common types of flip-flops. Like all Flip Flops, it has the ability to retain one bit of digital information. D flip-flop is applicable for synchronous circuits. In this paper NSGA, NSGA-II, and proposed FNSGA-II are employed to find the best channel widths and supply voltage in which the D Flip-Flop has the lowest average power and propagation delay of proposed dual edge-triggered static D flip-flop circuit. This study is the further research of the previous article which was the single objective optimization of JK Flip-Flop layout sizes based on single objective optimization algorithms such as Ant Colony Optimization in Real or continuous domain ACOR, Fuzzy-ACOR, Genetic Algorithm GA, and Fuzzy-GA in which one objective function, the average power, was considered for minimization (Keivanian et al. 2014a).

## Proposed dual-edge triggered D flip-flop

**Fig. 1:**
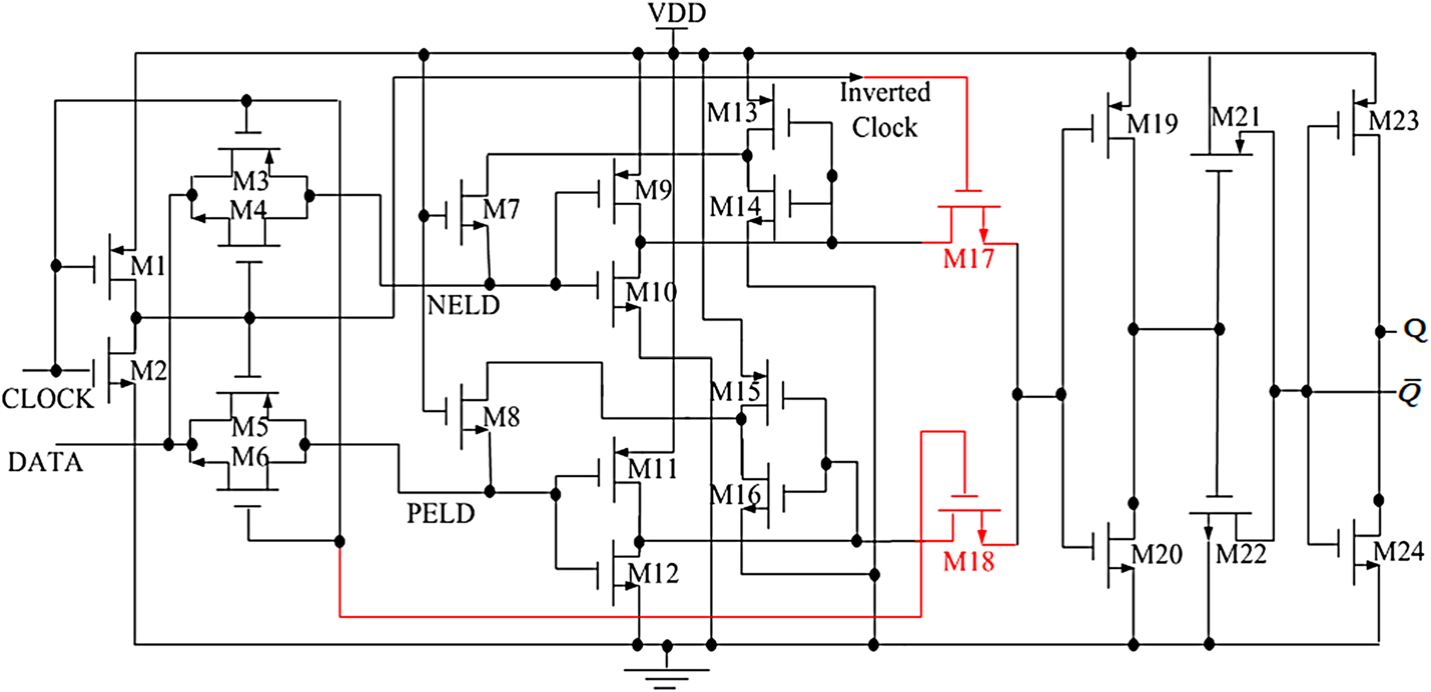
Proposed dual edge-triggered D flip-flop

The proposed dual-edge triggered static D Flip-Flop is shown in Fig. 1.

Overall, the operation of the circuit is to select input DATA and pass it on the output channel, Q. As it is illustrated in Fig. 1, the circuit is a synchronous multiplexer that can transmit multiple data simultaneously to output Q based on both edges of CLOCK pulse. In close view, to analyse the performance of circuit, two NMOSs of M17 and M18 were connected to each inverter module (one is M13 and M14, the other is M15 and M16) in order to boost their outputs. Back to back connected inverters keep the data when transmission gate is off. At the same time multiplexer transmits this latched data to the inverter to pass the correct DATA on the output line Q. Based on Fig. 1, when the CLOCK is low the MOSFETs M3, M4 and M18 are all on while M5, M6 and M17 are all off. Hence DATA is hold by negative latch and is passed to output line Q. In contrast, whenever CLOCK is high then the MOSFETs M5, M6 and M17 will be on but the MOSFETs M3, M4 and M18 will be off. In this state, DATA is passed on the output channel Q. So that, in dual edge-triggered D flip-flip DATA is put forward to output through both low and high states of CLOCK. Before the next CLOCK, if DATA alters this new amount of DATA is held by positive edge latch data PELD part and whenever next CLOCK comes and changes from Low to High this DATA is conveyed to the output channel Q. On the contrary, before the following CLOCK, if DATA changes this new DATA is hold by Negative Edge Latch Data NELD part and once next CLOCK arrives and alters from High to Low the DATA reach to the output channel Q. Without using M19, M20, M21, M22, M23, and M24 the output does not reach to the standard value of high or low level and there will be some transient time states for output signals. They should be a series of standard pulses since the input data is in fact a series of standard pulses.

**Fig. 2:**
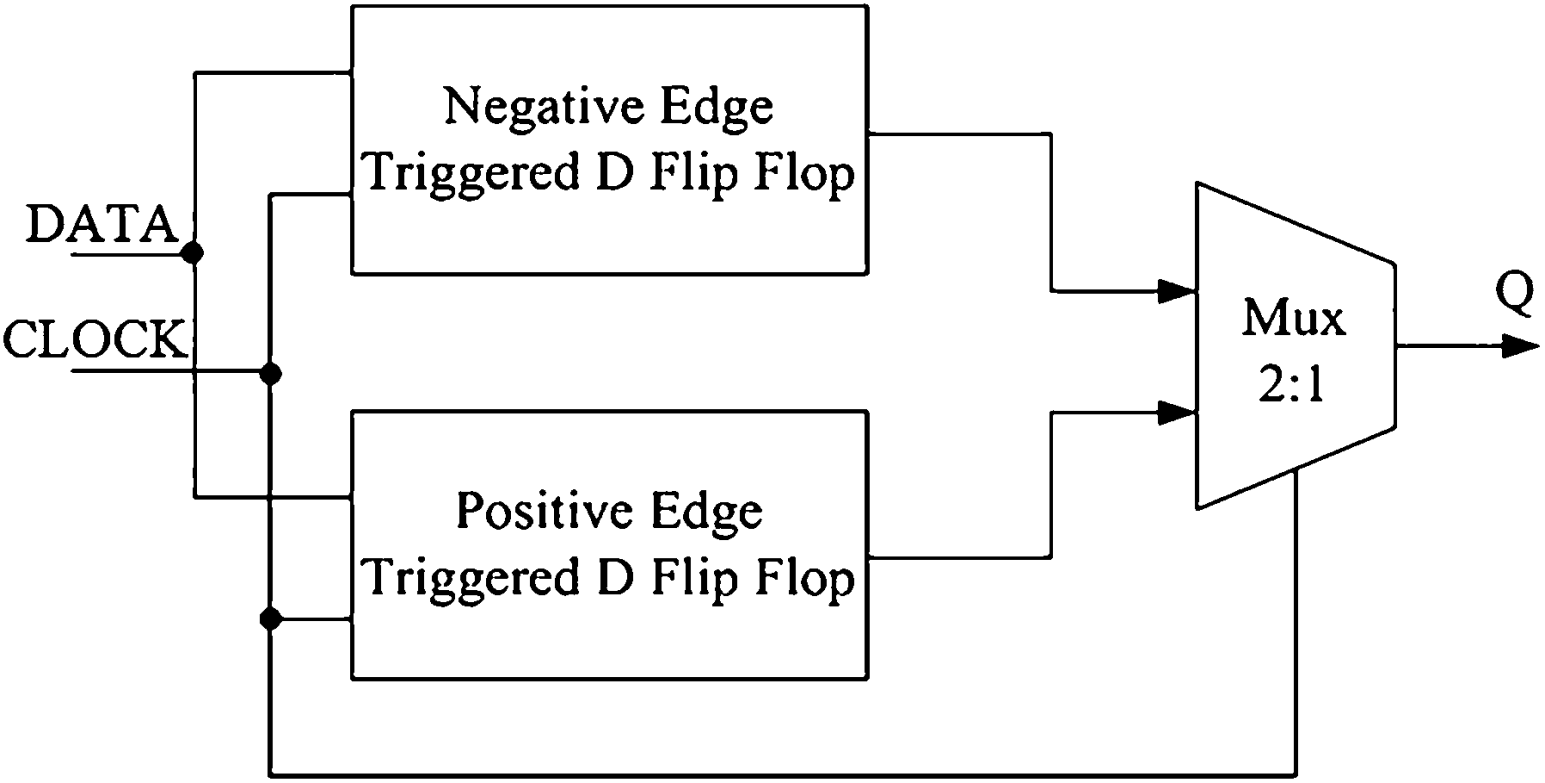
Dual-edge-triggered flip flops

The general configuration of multiplexer is shown as the block diagram in Fig. 2 (Nedovic et al. 2002).

**Fig. 3:**
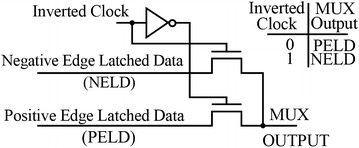
The 2:1 multiplexer with NMOS pass-transistor

Both positive and negative edges are used to sample the DATA at both edges of CLOCK and the appropriate sample is selected for the output Q by a clocked multiplexer, MUX. By using the double edge clocking the power in the CLOCK distribution network is saved. Base on Fig. 2, data is captured or sampled by both edges of the CLOCK also the appropriate sample is selected for the Q output (Singh and Sulochana 2013). In this architecture, the Multiplexer is designed by using two NMOS transistors as pass transistors that select either the positive edge or negative edge latched data to pass it to output channel based on Fig. 3 (Keivanian et al. 2014b).

We have proposed a new architecture in this literature that is dual edge-triggered Flip-Flop with NMOS pass-transistors as Multiplexer. In which the DATA can be passed by both positive and negative edges of CLOCK. This is more efficient in term of speed compared with single edge triggered Flip-Flop where DATA can only pass to output channel in a single triggering state of CLOCK (Singh and Sulochana 2013). In study, the design and performance parameters of Dual Edge-Triggered D Flip-Flop circuit to define it as an optimization problem are defined as in Table 1.

**Table 1:** The design and performance parameters of dual edge-triggered D flip-flop in this article

Design parameters	Performance parameters
Supply voltage (V_DD_)	Total average power (P_t_)
PMOS channel width (W_PMOS_)	
NMOS channel width (W_NMOS_)	Propagation delay time (t_PD_)

In this article all the channel lengths are set as the fixed value and equal to 0.18 micron L = 0.18 µm, whereas the channel widths are defined as the design parameters in circuit layout design literature and as the decision variables in meta-heuristic based optimization algorithms’ literature.

## Single-objective optimization

The minimization of average power Pavg (w) is addressed to single objective optimization problem and many techniques are demonstrated in this literature (Keivanian et al. 2014a, b; Keivanian 2014). For example, for single objective optimization of JK flip flop layout sizes the least dynamic average power obtained was 1.6 nw. But the propagation delay was not considered for optimization as a result the layout sizes could not provide the optimum speed for the circuit. This encouraged us to study more on the multi-objective optimization algorithms and the design and performance parameters of proposed dual edge-triggered static D flip-flop circuit in order to minimize its dynamic average power dissipation and propagation delay.

## Multi-objective optimization

Although single-objective optimization problems may have a unique optimal solution, multi-objective optimization problems, MOPs present a possible uncountable set of solutions, which when evaluated, produce vectors whose components represent trade-offs in objective space. Here the objective space is two dimensional including power consumption and delay objectives. In multi-objective optimization area a decision maker finally chooses an acceptable solution or solutions by selecting one or more of the solutions (Coello Coello et al. 2007). In this research work, the decision maker in fact is the electronic designers who evaluate the conditions and choose a candidate solution from the obtained set of solutions that it will have a minimum power delay product value.

The vector of decision variables in the Multi-objective optimization problem is found and satisfies the constraints and optimizes the objective functions (Coello Coello et al. 2007). These functions form a mathematical description of performance of problem which are usually in conflict with each other. In this article there is conflict between propagation delay and dynamic power dissipation (Singh and Sulochana 2013). The design parameters of problem are discovered to find optimum power consumption with reasonable delay time. So both will not be ideally obtained and a trade-off between them is required. Hence, the term “optimizes” means finding such a solution which would give the values of all the objective functions acceptable to the decision maker (Coello Coello et al. 2007).

In this article, our goal is to achieve a candidate solution for layout sizes and power supply values of circuit that will lead to a circuit with 6.32 PJ power delay product. So we firstly try to obtain optimum set of solutions with good performance then select a candidate solution from them with PDP = 6.32 PJ.

### Decision variables

The decision variables are the numerical quantities or control parameters of an optimization problem. In this article these quantities are denoted as x_i_, i = 1, 2, 3. T stands for transpose. Then the vector x with 3 decision variables is represented by the relation (1):

1 $$X^{T} = \left[ {V_{DD} \quad W_{PMOS} \quad W_{NMOS} } \right]$$

### Constraints

**Fig. 4:**
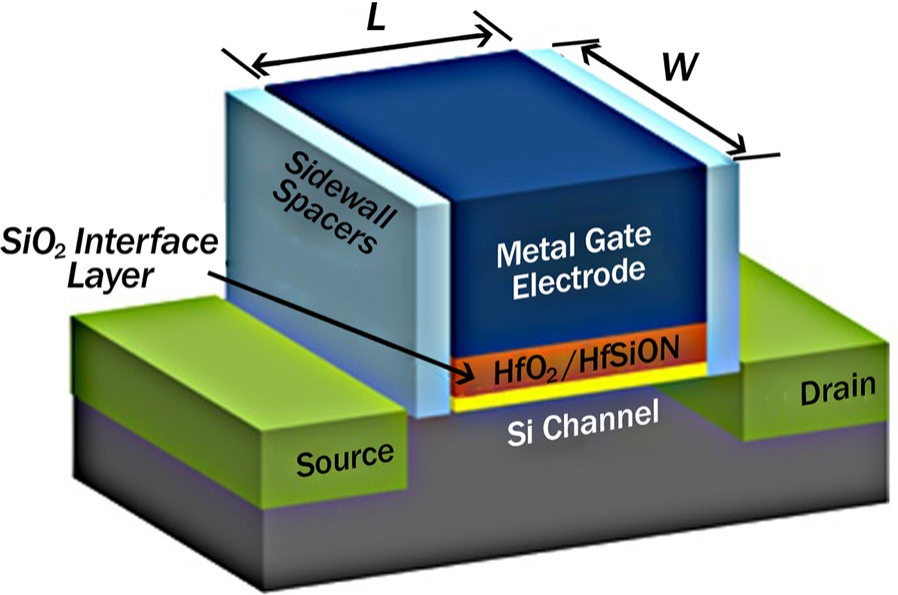
The channel width (W) and the channel length (L)

In most optimization problems some restrictions are proposed because of particular characteristics or physical limitations. In this study, the channel length is selected smaller than the channel width based on the relations (2) and (3). If the channel length L is selected as larger value of the channel width W, any change in W along the channel length will be too small compared with the channel width. Therefore, the electric field in the depletion region of the gate junction is assumed perpendicular to the channel (i.e., along the y-direction), while the electric field inside the neutral n-channel may be assumed to be in the x-direction only. The channel width w and channel length L both are figured out in Fig. 4.

The channel length L is from the source to drain, and it is a fixed parameter L = 0.18 µm. As the size of MOSFETs continues to scale down, the channel length becomes equal to or less than the depletion layer width of the source and drain junctions, and hence long-channel behavior occurs in short-channel devices(Li 2006).

In this article the following restrictions must be satisfied to meet the physical requirements of MOSFETs. All these restrictions are in general named, the **constraints**, and they show also the dependencies of decision variables **x** and constants involved in the problem, as in (2) and (3):

2 $$1\,\upmu{\text{m}} \le W_{NMOS} ,W_{PMOS} \le 1.8\,\upmu{\text{m}},\quad 1v \le V_{DD} \le 1.8v$$

3 $$L = 0.18\;\upmu{\text{m}} < W_{NMOS} ,W_{PMOS}$$

### Objective functions

The objective functions f_1_ (**w**) and f_2_ (**w**), form a vector function f (w) which is defined by (4):

4 $$f\left( {\mathbf{w}} \right) = \left[ {f_{1} \left( {\mathbf{w}} \right),f_{2} \left( {\mathbf{w}} \right)} \right]^{T}$$

There will not one unique solution instead a set of solutions will be produced which are based on the Pareto Optimality Theory (Ehrgott 2006).

### Dependency between total average power, channel widths, and supply voltage

Three major sources of power dissipation in CMOS VLSI circuits are dynamic, static, and the power based on leakage currents that are calculated in (5) to (7).

5 $$P_{Dynamic} = V_{DD}^{2} \cdot C \cdot f_{clock} \cdot N$$

In Eq. (5), N is the number of bits that are transmitted at a time, C represents the total capacitance which is the sum of internal capacitance of the circuit and the load capacitance at the output node. Also VDD is the power supply and f_clock_ is the frequency of the clock (Lyer 2010). The dynamic power is calculated only for switching capacitive power but static power and short-circuit power must be computed separately (Knepper 2009). The dynamic average power contributes the highest power consumption among others (Singh and Sulochana 2013). When the transistors are not in switching process, the static power is calculated:

6 $$P_{Static} = V_{DD} \cdot I_{DC}$$

In Eq. (6), I_DC_ is the total DC leakage current that is drawn from the power supply to the circuit (Chen et al. 2002). The Leakage Power calculation is presented in (7):

7 $$P_{Leakage Power} = V_{DD} \cdot I_{peak} \left( {\frac{{t_{r} + t_{f} }}{2}} \right)f_{clock}$$

In (7), I_peak_ is the peak or maximum transient current when the output node voltage is rising from threshold voltage V_T_ to V_DD_ − V_T_ or it is falling from V_DD_ − V_T_ to V_T_. Total average power is:

8 $$Total\;Power = P_{Dynamic} + P_{Static} + P_{Leakage Power}$$

In relation (8) all power values are added together for the calculation of total average power in a CMOS integrated dual edge-triggered static D flip-flop circuit. In order to get an accurate measurement for total average power, the appropriate range of time is determined for running a transient simulation in HSPICE software. Since the measurement involves an average of the instantaneous power value over the simulation window, selection of too small or too large transient simulation length may give inaccurate value (Wallace 2006). In this article, 9 CMOS inverters or 18 MOSFETs, two transmission gates, two NMOS pass transistors, and other MOSFETs constitutes the different transitions or time operations, thus for averaging of total power the simulation length will be as:

9 $$Simulation\;Window = 15 \times T_{Clock} = 15 \times 8\,ns = 120\,ns$$

In relation (9) the simulation window has the size of 15 × Period in second scale. This will be helpful to achieve an accurate average of total power. The corresponding command statement in HSPICE is:

10 $$.MEAS\;tran\;AvgPower\;Avg\;Power\;from = 0\,ps\;to = 120\,ns$$

Based on command (10), total average power is measured in the simulation window of 120 ns.

11 $$Total\;Average\;Power = \frac{1}{120\,ns}\int\limits_{0}^{120\,ns} {\left[ {P_{Dynamic} + P_{Static} + P_{Leakage Power} } \right]} \,dt$$

In Eq. (11), the dynamic power *P*_*Dynamic*_ is the highest power consumption among the other consumption powers. Also based on (5), P_Dynamic_ value depends on squared power supply V_DD_^2^; so reducing the supply voltage is the most effective way for reduction of the Total Average Power of the circuit. However it will decrease the speed of the circuit that is explained in the next part as 2nd objective function. Reduction in clock frequency is another alternative to reduce the dynamic power. Double edge clocking approach is adapted in this paper to reduce the clock frequency.

12 $$Total\;Average\;Power \propto V_{DD}^{2}$$

The relation (12) shows that there is a direct relation between V_DD_^2^ and total average power P_t_.

Also the total capacitance, C in relation (5) and the rise or fall time in relation (7) all depend on channel widths in each inverter (Stiles 2014) that is used in the circuit:

13 $$C \approx C_{{ox^{ \cdot } }} \left( {W_{{P^{ \cdot } }} + W_{N} } \right) \cdot L$$

The Eq. (13) indicates that the parameter C relates directly to PMOS and NMOS channel widths.

14 $$\frac{{t_{rise} }}{{t_{fall} }} = \frac{{I_{DN\;\hbox{max} } }}{{I_{DP\;\hbox{max} } }} = \frac{{\mu_{n} }}{{\mu_{p} }} \cdot \left[ {\frac{{W_{NMOS} \cdot \left( {V_{DD} - V_{THN} } \right)}}{{W_{PMOS} \cdot \left( {V_{DD} - \left| {V_{THP} } \right|} \right)}}} \right]^{2}$$

The ratio (14) shows that any changes in the channel widths will affect the rise and fall time in each inverter (Stiles 2014). Consequently:

15 $$Total\;Average\;Power \propto W$$

The relation (15) shows the direct relation between the channel widths and the Total Average Power.

### Dependency of propagation delay time t_PD_ to the channel widths W and V_DD_

**Fig. 5:**
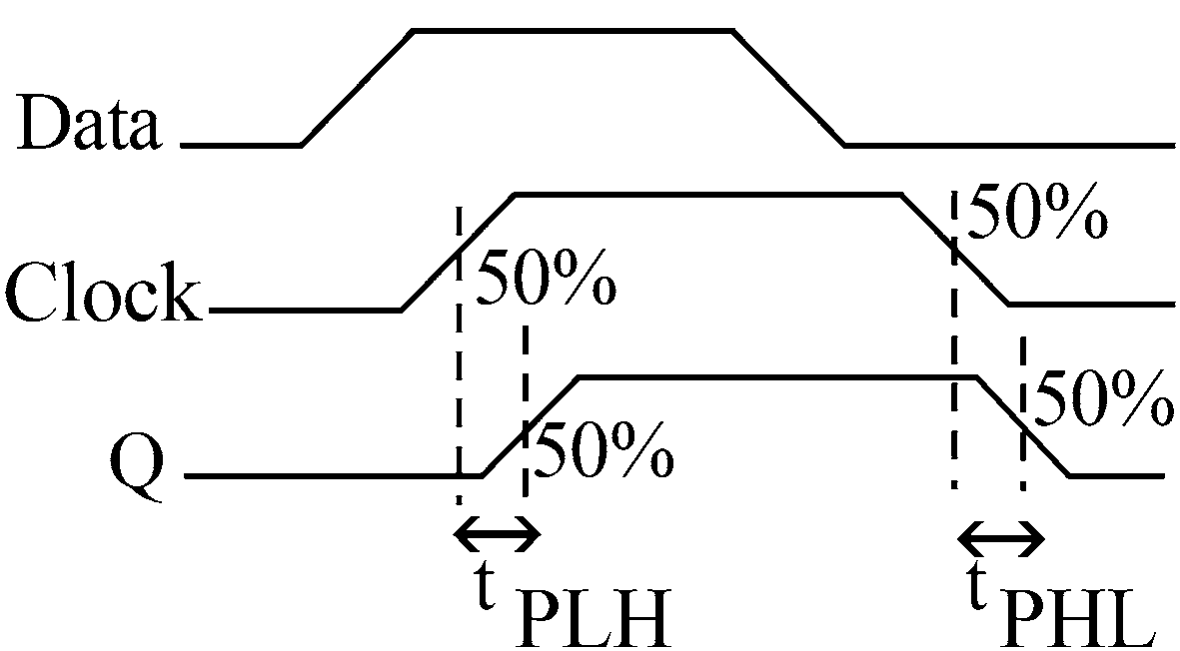
Propagation delay time (t_PHL_ and t_PLH_)

Propagation delay time is the time taken from the triggering input transition to the corresponding output transition. The transitions are measured from the 50 % point. The output node Q is measured relatively to the input clock pulse as it is shown in Fig. 5.

The propagation delay time is calculated by (16):

16 $$t_{PD} = \frac{{t_{PHL} + t_{PLH} }}{2}$$

In relation (16), t_PD_ is the propagation delay time and inappropriate value for it may cause timing problems in while system. In detail view, t_PHL_ and t_PLH_ in inverters are measured by (17)

17 $$\begin{array}{*{20}l} {t_{PHL} = \frac{{C_{load} }}{{K_{n} \cdot \left( {V_{DD} - V_{TH,n} } \right)}}\left[ {\frac{{2 \cdot V_{TH,n} }}{{V_{DD} - V_{TH,n} }} + \ln \left( {\frac{{4\left( {V_{DD} - V_{TH,n} } \right)}}{{V_{DD} }} - 1} \right)} \right]} \hfill \\ {t_{PLH} = \frac{{C_{load} }}{{K_{p} \cdot \left( {V_{DD} - \left| {V_{TH,p} } \right|} \right)}}\left[ {\frac{{2 \cdot \left| {V_{TH,p} } \right|}}{{V_{DD} - \left| {V_{TH,p} } \right|}} + \ln \left( {\frac{{4\left( {V_{DD} - \left| {V_{TH,p} } \right|} \right)}}{{V_{DD} }} - 1} \right)} \right]} \hfill \\ \end{array}$$

In Eq. (14), K_N_ and K_P_ are the Trans Conductance parameters that are determined through technological properties that are used for fabrication of integrated circuits (µA/V^2^) (Stiles 2014). C_load_ is the load capacitance of inverters in the circuit, and V_DD_ is the supply voltage. The dependency of V_DD_ and t_PD_ is presented in (18):

18 $$\Pr opagation\;Delay\;Time \propto \frac{1}{{V_{DD}^{2} }}$$

The relation (15) shows that the propagation delay time is inversely proportional to the squared supply voltage; therefore increasing the supply voltage is the most effective way to reduce the Propagation Delay Time.

When the supply voltage value is increased, the charging current of the switching capacitances in the circuit is increased this will decrease the propagation delay through the logic, so the maximum frequency of the circuit or the maximum speed of flip-flop is increased (Varnes 2013).

Moreover in the transient conductance parameters are shown in (19):

19 $$\begin{array}{*{20}l} {K_{n} = \mu_{n} \cdot C_{ox} \cdot \left( {\frac{w}{L}} \right)} \hfill \\ {K_{p} = \mu_{p} \cdot C_{ox} \cdot \left( {\frac{w}{L}} \right)} \hfill \\ \end{array}$$

The Eq. (19) presents that the transient conductance parameters are dependent on the channel widths. Based on the relations of (16), (17) and (19) the resulting relation is:

20 $$t_{PD} \propto \frac{1}{\text{w}}$$

The relation (20) indicates that there is a reverse relation between channel width w and the total propagation delay (t_PD_).

## Implementations in HSPICE software

The command statements of HSPICE (.sp file) are including: 1. Clock (Low level = GND, High level = VDD, pulse width = 4 ns, and period time = 8 ns), 2. Input Data (“1111010110010000” with 7.5 ns time duration for each bit, Low level = GND, and High level = VDD), 3.Selection the MOSFETs model, and 4.Transient Analysis and Measurement of Total Average Power and Propagation Delay Time.

### MOSFET model for proposed dual edge-triggered-static D flip-flop

In Simulation Program for Integrated Circuit Engineering, SPICE, the models are defined for MOSFET devices. These models can be divided into three groups: (a) First Generation Level 1, Level 2, and Level 3 Models, (b) Second Generation BISM, HSPICE Level 28, BSIM2 and Third Generation Models, and (c) BSIM3, Level 7, Level 49 … models. The state-of-art models have better performances concerning the short channel effects, local stress, transistors’ operation in the sub-threshold region, gate Leakage tunneling, noise calculations, and temperature variations. In these new models the equations can converge better during circuit simulations (Lynn Fuller 2011). The level 49 model is the enhanced version of BSIM3v3. This compliance includes numerically identical model equations, and range limit parameters.

Through the DC model comparisons it is concluded that Third generation MOSFET models such as Level 7 for OrCAD/PSPICE or Level 49 models for HSPICE give better results than any of the first or second generation models.

The level 49 BSIM3 Version 3 MOS Model is originated from UC Berkeley and it has been installed as Level 49 in HSPICE software (Moon 1998). The performance of level 49 has been improved by reducing the complexity of model equation, replacing some calculations with spline functions, and optimizing the compiler. The simulation results will have time reduction up to 35 % (Star-HSPICE Manual-Release 2001).

In this article, we have used BSIM3v3 LEVEL = 49, VERSION = 3.22 model for NMOS and PMOS MOSFETs.

### Link between HSPICE and MATLAB

**Fig. 6:**
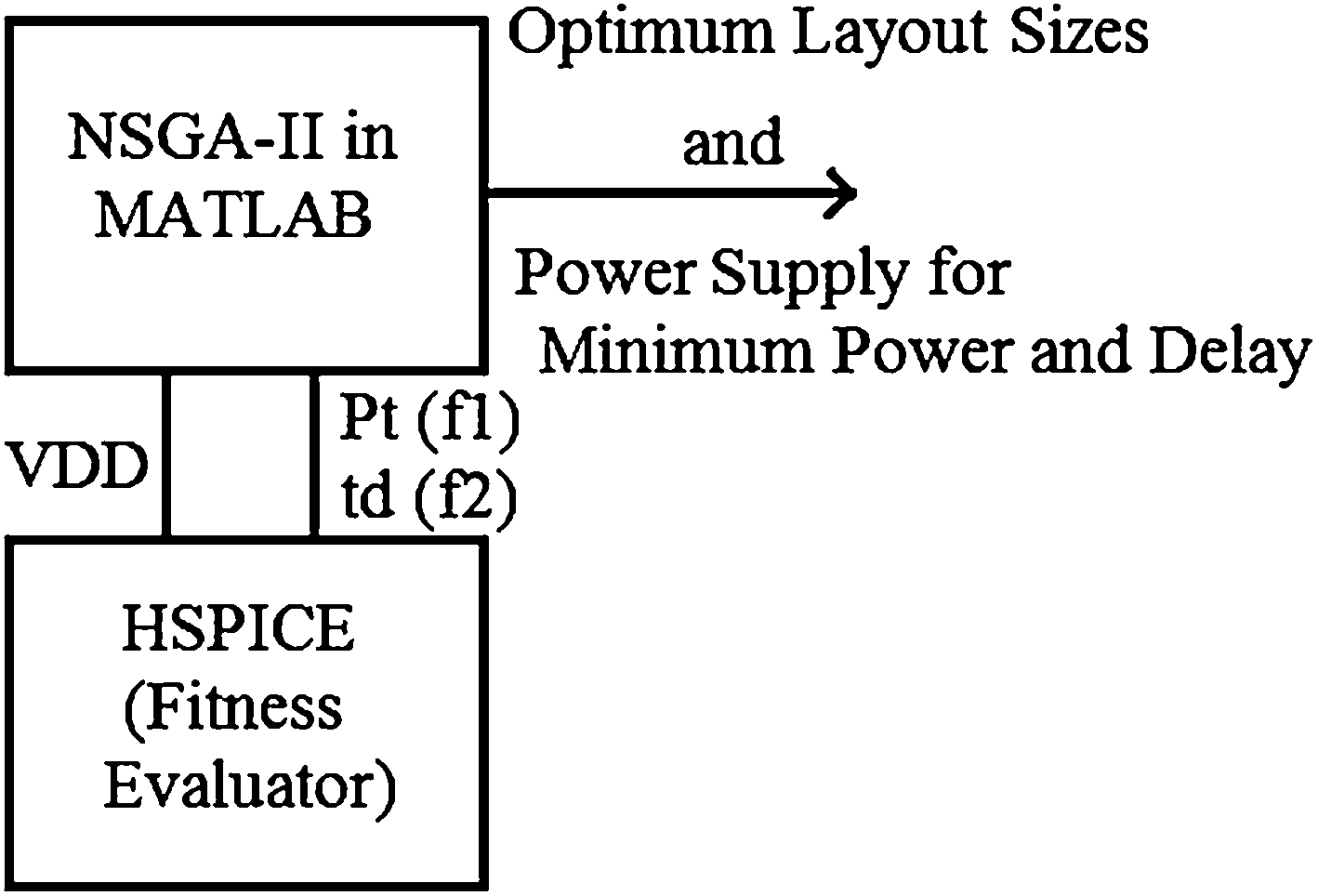
The block diagram of link between MATLAB and HSPICE

The decision variables V_DD_, W_PMOS_, and W_NMOS_ are altered by means of mutation, crossover, and selection procedures in NSGA-II in MATLAB software and the result values are printed in HSPICE file for example ‘D.sp’. Then HSPICE or namely the fitness evaluator is run to read the netlist file like ‘D.lis’ related to two objective function values including Total Average Power P_t_ and Propagation Delay Time t_D_. The best values for them are stored and the algorithm continues until the stopping criteria that is the maximum iteration number. Both HSPICE and MATLAB software are implemented simultaneously and the results are shared with them. Based on Fig. 6 in each of implementations MATLAB will produce the layout sizes of MOSFETs in dual edge-triggered D flip-flop circuit W_PMOS_, W_NMOS_, and the supply voltage value V_DD_ for HSPICE to simulate the proposed circuit by using these design parameters. Then the total average power and the propagation delay time correspondence to the circuit are evaluated by HSPICE. This implementation continues until the least average power and propagation delay time is obtained.

## Implementation of MATLAB

**Fig. 7:**
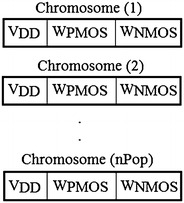
The chromosomes and genes in NSGA-II for solving the proposed problem

As it is shown in Fig. 7 each chromosome includes three genes. These are the design parameters of proposed problem. Concerning the number of population, there are twenty-seven 27 chromosomes each one has three genes. The NSGA-II operators are applied to them.

### Initial setting of the parameters in NSGA-II

The parameters of mutation, crossover, selection, and population size are in Table 2.

**Table 2:** Initial setting of the parameters in NSGA-II

Number of decision variables	3
Maximum iteration	50
Population size	27
Crossover percentage	0.7
P single point	0.1
P double point	0.2
Mutation percentage	0.4
Mutation rate (mu)	0.02

As stated in Table 2, the crossover and mutation percentage are determined at the first part of algorithm, they determine the number of parents and mutants respectively as follow:

21 $$N_{cross} = 2 \cdot round\left\{ {{{Crossover\;Percentage \times Pop} \mathord{\left/ {\vphantom {{Crossover\;Percentage \times Pop} 2}} \right. \kern-0pt} 2}} \right\}$$

22 $$N_{Mutation} = round\left\{ {Mutation\;Percentage \times Pop} \right\}$$

The mutation step value is calculated by (23)

23 $$Sigma = 0.1\left( {Var_{\text{max} } - Var_{\min} } \right)$$

By considering the constraints of (2) and (3), the design parameters are generated within the interval [Var_min_ Var_max_]. These maximum and minimum values are inspired by physical limitations of circuit. The *Sigma* value affects the exploration capability of the algorithm and it is named the mutation step. The coefficient is less than one to decrease the computational time and keeps a reasonable exploration capability for algorithm.

Two parameters Single and Double points as the distribution indexes are in (24):

24 $$pUniform = 1 - P\;Single\;Point - P\;Double\;Point$$

The applied method for the selection step is Roulette Wheel Selection. The index number generated by the *Roulette Wheel Selection function* in (25) determines the method of crossover:

25 $$M = Roulette\;Wheel\;Selection\left( {pSinglePoint,\;pDoublePoint,\;pUniform} \right),\quad 1 \le M \le 3$$

M is set one or two or three. One refers to single point crossover, two refers to double point crossover, and three refers to uniform distribution crossover type.

## Non-dominated sorting genetic algorithms: NSGA and NSGA-II

In the literature of non-dominated sorting Genetic Algorithm, there is NSGA approach that was relatively successful during several years for example in Coello Coello et al. (2007), and Reed et al. (2001), in this study we also implement and apply it for optimization of our problem.

### Performance measurement: *r*

In order to investigate how well the algorithms have distributed solutions over the non-dominated region, we use the Chi square-like deviation form distribution measure used elsewhere (Srinivas and Deb 1994).

26 $$r = \sqrt {\sum\nolimits_{i = 1}^{q + 1} {\left( {\frac{{n_{i} - \bar{n}_{i} }}{{\sigma_{i} }}} \right)^{2} } }$$

where *q* is the number of desired optimal points and (*q* + 1)-th sub-region is the dominated region, *n*_*i*_ is actual number of solutions serving i-th sub-region (niche) of the non-dominated region, $\bar{n}_{i}$ is expected number of solutions serving i-th sub-region of the non-dominated region, and *σ*_*i*_^2^ is the variance of solutions serving i-th sub-region of the non-dominated region. Using probability theory Deb estimated *σ*_*i*_^2^ value by (27):

27 $$\sigma_{i}^{2} = \bar{n}_{i} \cdot \left( {1 - \frac{{\bar{n}_{i} }}{P}} \right)$$

where *P* is the population size, therefore, an algorithm with a good distributing capacity is characterize by the lower deviation value and performance measure *r*.

## Proposed fuzzy NSGA-II algorithm: FNSGA-II

The mutation and crossover rates can be changed adaptively during the implementation runs. In this part, a fuzzy inference system FIS is proposed to balance between exploration and exploitation capabilities of NSGA-II algorithm, so the *Mutation Percentage* and *Crossover Percentage* are updated in each iteration step. They will determine the exploration and exploitation respectively.

Some fuzzy rules are defined in FIS system that are fired based on input values, as in Table 3.

**Table 3:** The fuzzy rules in FIS system

Rules	1	2	3	4	5
If	It is low and *r* is high	It is low and *r* is low	It is low and *r* is medium	It is medium and *r* is medium	It is high and *r* is low
Then	Mutation is high, crossover is low	Mutation is low, crossover is high	Mutation is high, crossover is low	Mutation and crossover medium	Mutation is low, crossover is high

**Fig. 8:**
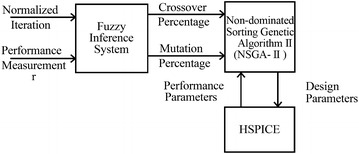
The block diagram of fuzzy inference system combined with NSGA-II in FNSGA-II algorithm

Along with the iteration runs, the algorithm may get stuck in a local solution or position, in this case the mutation percentage should be increased and the crossover should be decreased so the idea comes up to propose a fuzzy inference system based on input and output variables in this problem that includes the fuzzy rules in Table 3. Also there may be some solutions that are good but in the first iterations of algorithm, in this condition the crossover should be increased and mutation ought to be reduced. The configuration of Fuzzy NSGA-II algorithm is illustrated in Fig. 8.

## Implementation results

Concerning the performance measurement *r* the lower value for it will lead to the better distribution of solutions and for the proposed problem in this article, the electronic engineers will have more flexibility in circuit design. Table 4 presents the performance measure *r* multi-objective in the literature of non-dominated sorting genetic algorithm like NSGA, NSGA-II, and the newly proposed FNSGA-II in this article.

**Table 4:** Performance measure *r*

Algorithm	Performance measure *r*
NSGA	2.5
NSGA-II	2.3
FNSGA-II	2

The measurement results show that the proposed Fuzzy NSGA-II outperforms the other comparing algorithms therefore we apply it for optimization of layout sizes and power supply in dual edge-triggered static D flip-flop circuit.

We have selected one solution with lower PDP among the first Pareto Front solutions for three algorithms to compare them. We can see that the performance of algorithm NSGA-II is improved by Fuzzy Inference System because of well trade-off between mutation and crossover processes. The candidate obtained power delay product PDP for FNSGA-II is the best one, 6.32 PJ. This goal is achieved when the design parameters are set based on Table 5 values (V_DD_ = 1.21 v, W_P_ = 1.27 μms, and W_N_ = 1.01 µm).

**Table 5:** Candidate Solutions obtained by algorithms

Algorithm	Design parameters	Performance parameters	Power delay product (PDP) (PJ)
FNSGA-II	V_DD_ = 1.17 v	Total average power P_t_ = 172 µw	6.32
	W_PMOS_ = 1.37 µm		
	W_NMOS_ = 1.02 µm	Propagation delay t_d_ = 3.676e−08	
NSGA-II	V_DD_ = 1.21 v	Total average power P_t_ = 175 µw	6.65
	W_PMOS_ = 1.27 µm		
	W_NMOS_ = 1.01 µm	Propagation delay t_d_ = 3.8e−08	
NSGA	V_DD_ = 1.30 v	Total average power P_t_ = 180 µw	7.20
	W_PMOS_ = 1.15 µm		
	W_NMOS_ = 1.00 µm	Propagation delay t_d_ = 4e−08	

## Conclusion

 A new dual edge-triggered static D Flip-Flop with two NMOS MOSFETs is proposed. The design parameters including NMOS/PMOS channel widths and power supply V_DD_ and performance parameters such as average power consumption and delay are investigated. The required background mathematics showed the relationships between them. So a black box of multi-objective optimization algorithm can be defined because the input and output variables are clarified. We then proposed a fuzzy inference system FIS that contains some fuzzy rules. They are fired during the iteration steps to adaptively tune the exploration and exploitation parameters of proposed Fuzzy Non-dominated Sorting Genetic Algorithm, NSGA-II. The literature showed that the two parameters of GA, *P*_*mutation*_, and *P*_*crossover*_ may significantly influence the performance of the algorithm. FNSGA-II handles this problem by performing an automatic adaptation of the two parameters of GA taking into account both the global and local optimization, thus diminishing the problem of falling into local minima. The exploration parameters are decreased during the execution of FNSGA-II aiming to quickly find the optimal solution. The iterative link between MATLAB’s algorithm and HSPICE layout design circuit is continued until the stopping criteria, the maximum iteration. Finally FNSGA-II proposed in this paper enables finding solutions that are better distributed the region of solutions of two objective functions. The layout design of suggested Dual Edge-Triggered Static D Flip-Flop Circuit is completed because the optimum values for PMOS and NMOS channel widths W_PMOS_ and W_NMOS_ also the optimum amount for power supply value V_DD_ are obtained by FNSGA-II and base on these values the circuits met the minimum average power and propagation delay time. The power delay product PDP became 6.32 PJ that is good for critical design sensitive to the time and power.
